# The transcription repressor Bach2 is required for maintaining the B-1 cell population by regulating self-renewal

**DOI:** 10.3389/fimmu.2025.1553089

**Published:** 2025-03-18

**Authors:** Seung-Gen Oh, Jeonghyun Noh, Eunkyeong Jang, Jeehee Youn

**Affiliations:** ^1^ Department of Biomedical Science, Graduate School of Biomedical Science and Engineering, Hanyang University, Seoul, Republic of Korea; ^2^ Laboratory of Autoimmunology, Department of Anatomy and Cell Biology, College of Medicine, Hanyang University, Seoul, Republic of Korea

**Keywords:** B-1 cells, BACH2, self-renewal, B-1 cell reconstitution, conditional knockout mouse model

## Abstract

B-1 cells are a distinct lineage of tissue-resident B cells with crucial roles in innate immunity and tissue homeostasis. Mature B-1 cell pools are mostly maintained by self-renewal in their peripheral niches, in a process that is largely uncharacterized. Here, we investigated the role of the transcription repressor Bach2 in maintaining the B-1 cell pool. We found that B-1 cell numbers and antibody responses were dramatically reduced in adult mice bearing a B cell-specific *Bach2* deletion, although the proportions of B-1 progenitors in early neonatal life were unaffected. Cells taken from the fetal liver or bone marrow of *Bach2*-deleted mice were defective in reconstituting the B-1 cell pool in the peritonea of *Rag2^-/-^* hosts, and peritoneal B-1 cell transplants from adult *Bach2*-deleted mice failed to sustain their numbers in the host’s peritoneum. The mutant B-1 cells proliferated normally *in vivo* but were more apoptotic. They also expressed the reduced level of the self-renewal factor Bmi1. These results indicate that Bach2 deficiency does not affect the development of B-1 progenitors in fetal liver and bone marrow but impairs the self-renewal of mature B-1 cells in peripheral tissues, which is caused by increased apoptosis. Thus, this study suggests that a cell-autonomous function of Bach2 is crucial for maintaining a stable population size of B-1 cells in their peripheral niches.

## Introduction

B-1 cells are a unique subset of B cells with innate-like properties, which are distinct from conventional B-2 cells in their origin, locations, and functions. They are defined as CD19^hi^B220^lo^IgM^hi^CD23^-^CD43^+^IgD^lo^ cells, and are further subdivided into major CD5^+^ B-1a and minor CD5^-^ B-1b subsets, although the identity and origin of the latter are subjects of considerable debate (1, 2). B-1 cells predominantly reside in pleural and peritoneal cavities where they constitute 35-70% of the local B cells, while they make up 1-2% of the B cell fraction in the spleen and bone marrow (BM) (2). They are a main source of natural antibodies (Abs) at steady state, as evidenced in allotype chimeric mice in which at least 80% of the serum IgM is derived from B-1 cells (3). Several studies indicate that natural IgM-secreting B-1 cells are primarily found in the spleen and BM rather than in the pleural and peritoneal cavities (4–6). This suggests that activated B-1 cells in coelomic cavities migrate to the organs, where they further differentiate into Ab-secreting cells. In contrast to B-2 cells, B-1 cells are positively selected for binding to self-antigens and so develop a poly-specific BCR repertoire during early development (7, 8). This effect enables B-1 cell-derived Abs to broadly cross-react with microbial antigens and altered self-antigens, which underpins the innate-like roles of B-1 cells in maintaining tissue homeostasis as well as providing protective immunity.

The *de novo* development of B-1 cells mostly occurs before birth and during the first few weeks after birth. Accumulating evidence suggests that B-1 cells arise in multiple waves from progenitors distinct from B-2 cell progenitors. The first wave initiates in the yolk sac and splanchnopleura region in the early mouse embryo (9, 10). During the second wave, hematopoietic stem cells in the fetal liver take over as the main source of B-1 cells (11, 12). The third wave takes place in the BM, and is much less efficient (12, 13). Unlike B-2 cells, which are continuously replenished from the BM, B-1 cells are largely maintained by self-renewal once they have populated their various tissue locations (14, 15). Although BM B-1 progenitors generate a small number of B-1 cells *de novo*, B-1 cells are only very inefficiently reconstituted from this pathway in adult mice (13). To maintain the pools of B-1 cells, these cells themselves multiply homeostatically to replace dying cells and thus sustain a stable population size over time. Interestingly, B-1 cells in the peritoneal cavity have a longer lifespan and slower turnover rate than their B-2 counterparts (16). This long-term survival seems to be independent of BAFF and is associated with unique characteristics of intracellular signaling, including relative unresponsiveness to BCR-crosslinking and active basal tonic signaling downstream of the BCR (17–20). Moreover, B-1 cells are probably controlled by a transcriptional program that differs from that of B-2 cells. For example, the transcription factor Bhlhe41 has been shown to specifically control the development and self-renewal of B-1 cells (21), and Bmi1, a component of polycomb repressor complex 1, is required for maintaining B-1 cell pools but not B-2 cell pools (22). However, current understanding of the physiology of B-1 cells is rudimentary.

The transcriptional network that controls immune cell fate choices includes the basic region leucine zipper transcription repressor BTB and CNC homology (Bach)2. Bach2 seems to function in lineage commitment during BM hematopoiesis, as evidenced by the finding that common lymphoid progenitors and common myeloid progenitors differentiate preferentially into myeloid-lineage cells at the expense of B- and erythroid-lineage cells, respectively, in the BM of Bach2-deficient mice (23, 24). In addition to its role in the BM, Bach2 also controls mature B cell fates in the periphery. Repression by Bach2 of *Prdm1*, which encodes Blimp-1, supports germinal center (GC) formation and thereby prevents the premature emergence of IgM-secreting plasma cells (25, 26). The expression level of Bach2 is also crucial for directing GC B cell conversion into either plasma cells or memory cells (27). Collectively, these studies point to the importance of Bach2 in determining the fates of peripheral B-2 cells, but it has not been known whether Bach2 is involved in the transcriptional program for the development and/or self-renewal of B-1 cells.

Here, we report that the absence of Bach2 impaired generation of the mature B-1 cell pool more severely than that of the mature B-2 cell pool. The number of B-1 cells was dramatically reduced in mice in which Bach2 was specifically deleted in B-lineage cells. Detailed analysis established a major role for Bach2 in controlling the self-renewal of B-1 cells rather than their *de novo* development.

## Materials and methods

### Mice

Breeders of *Bach2^fl/fl^* mice (27) were kindly provided by Dr. Tomohiro Kurosaki (Osaka University, Japan). CD45.1, *Rag2^-/-^* and B6.129P2(C)-Cd19tm1(cre)Cgn/J (referred to hereafter as *Cd19^cre/+^*) mice on a C57BL/6 background were purchased from the Jackson Laboratory. *Bach2^fl/fl^* and *Cd19^cre/+^* strains were maintained in the animal facility of Hanyang University under specific pathogen-free conditions. B cell-specific Bach2-deficient (*Cd19^Cre/+^Bach2^fl/fl^*) conditional knockout (cKO) mice were generated by crossing *Cd19^cre/+^* and *Bach2^fl/fl^* mice, and their *Cd19^Cre/+^Bach2^fl/+^* littermates were used as wild-type (WT) counterparts. Age- and sex-matched mice were used in all experiments. In bromodeoxyuridine (BrdU) incorporation assays, mice were fed drinking water containing 0.8 mg/ml BrdU (Sigma-Aldrich) for 7 days. All protocols concerning animal use were approved by the Institutional Animal Care and Use Committee of Hanyang University, and all animal experiments were carried out in strict accordance with guidelines and regulations.

### Cell preparation

Peritoneal exudate cells (PECs) were collected from the abdominal cavity of mice by performing lavage three times, each time with 2 ml of PBS containing 1% fetal bovine serum (FBS). Any PECs contaminated with blood were discarded. Livers were removed from cKO or WT fetuses on embryonic day (E) 15.5. To obtain single-cell suspensions of splenocytes and fetal liver cells, spleens and fetal livers were ground, followed by osmotic lysis of erythrocyte, and filtered through a 70 μm nylon mesh strainer. BM cells were extracted from tibias and femurs by flushing and subjected to osmotic lysis to remove erythrocytes. In some experiments, CD19^+^ B cells among the PECs were isolated by magnetic-activated cell sorting (MACS; Miltenyi Biotec) to > 95% purity. The B-2, B-1a, and B-1b cells among the PECs were sorted with a FACS Aria III (BD Biosciences) to > 97% purity.

### Flow cytometry

Aliquots of mouse single cell suspensions were stained with appropriate combinations of monoclonal Abs (mAbs) and analyzed by fluorescence-activated cell sorting (FACS), as described previously (28). In brief, cells were first incubated with anti-CD16/32 mAb (2.4G2, Cat#553142, BD Biosciences) to block Fc receptors and then stained with fluorochrome- or biotin-conjugated mAbs in FACS buffer. Dead cells were excluded from the analysis using a Live/dead cell staining kit (Cat# L10120, L23101 and L10119, eBioscience). Intracellular staining to detect proliferating cells was performed using anti-Ki-67-PE (B56, Cat#567719, BD Biosciences) after treatment with Fixation/Permeabilization Solution (Cat# 554714, BD Biosciences). In BrdU incorporation assays cells were stained with mAbs for B220 and CD19, then fixed, permeabilized, treated with DNase. Subsequently, cells were stained with anti-BrdU-FITC mAb according to the manufacturer’s instructions (BioLegend). Anti-CD45.2-FITC (104, Cat#109806), anti-IgM-FITC (RMM-1, Cat#406506), anti-CD21/35-FITC (7E9, Cat#123408), anti-CD19-PE (6D5, Cat#115508), anti-CD19-PerCP (6D5, Cat#115531), anti-CD93-PE (AA4.1, Cat#136503), and anti-CD45.2-PE-Cy7 (104, Cat#109829) were purchased from BioLegend. Anti-streptavidin-FITC (Cat#554060), anti-B220-FITC (RA3-6B2, Cat#553088), anti-Annexin V-FITC (Cat#51-65874X), anti-CD117 (c-Kit)-PE-Cy7 (2B8, Cat#558163), anti-CD23-PE-Cy7 (B3B4, Cat#101614), anti-B220-APC-Cy7 (RA3-6B2, Cat#552094), anti-CD25-APC-Cy7 (PC61, Cat#557658), anti-BrdU-FITC (B44, Cat#347583), anti-Annexin V-APC (Cat#550475), 7-AAD (Cat#559925) and the Biotin Mouse Lineage Panel (Cat#559971) were from BD Biosciences. Anti-CD45.1-PE (A20, Cat#12-0453-81), anti-IgD-PE (11-26C, Cat#12-5993-82), anti-CD5-APC (53-7.3, Cat#17-0051-82), and anti-B220-APC (RA3-6B2, Cat#17-0452-83) were from Invitrogen.

### Generation of chimeric mice

Six-to-eight-week-old *Rag2^-/-^* mice were irradiated at a dose of 500 rad and injected intravenously with fetal liver or BM cells on the same day. To generate fetal liver chimeric mice, 2x10^6^ fetal liver cells from cKO or WT embryos on E15.5 were used, and to generate BM chimeric mice, 5x10^6^ BM cells from 8-10-week-old cKO or WT mice were used. Eight weeks after transplantation the recipients were assayed *post mortem* by FACS.

### Adoptive cell transfer

CD19^+^ B cells sorted from WT or cKO PECs were labeled with 3 μM carboxyfluorescein succinimidyl ester (CFSE; Molecular Probes). The cells (5x10^5^) were resuspended in 100 μl of PBS and transferred intraperitoneally into 10-week-old un-irradiated C57BL/6 mice. Two weeks later, PECs were obtained from the recipients and the fluorescence intensity of CFSE was determined by FACS. To transfer a mixture of WT and cKO B-1a cells, B-1a cells were isolated from CD45.1^+^ C57BL/6 and Bach2 cKO (CD45.2^+^) mice using a FACSaria III. A 1:1 mixture of the cells (total 2 × 10^5^ cells) was intraperitoneally injected into 500 rad-irradiated *Rag2^-/-^* recipient mice. Twelve weeks later, PECs were obtained from the recipients and analyzed by FACS.

### Immunization with T cell-independent antigen

Mice were injected intraperitoneally with 50 μg of (4-hydroxy-3-nitrophenyl)-acetyl (NP)-Ficoll (Biosearch Technology) in 100 μL of PBS. Six days after immunization, sera, peritoneal lavage fluid and spleens were collected *post mortem* and assayed by ELISA and ELISPOT.

### ELISA and ELISPOT

Titers of anti-NP IgM Abs and numbers of anti-NP IgM Ab-secreting cells (ASCs) were assessed by ELISA and ELISPOT assay, respectively (29). For ELISAs, immunosorbent plates were coated with 20 μg/ml NP_36_-BSA (Biosearch Technologies). Serum samples diluted 1:1000 and peritoneal lavage fluids without further dilution were used. Titers of total IgM (Alpha Diagnostic International) and phophorylcholine-specific IgM Abs were determined by ELISA in sera of six-week-old WT and Bach2 cKO mice, as previously described (30, 31).

### RT-quantitative PCR

Total RNA was purified from sorted subsets of B cells using TRIzol reagent (Invitrogen) and assayed as described (32). cDNA was synthesized with amfiRivert cDNA Synthesis Master Mix (GenDEPOT) and amplified by quantitative PCR using SYBR Green Quantitative PCR Master Mix (Applied Biosystems). The mRNA levels of each gene were normalized to that of β-actin, and differences between samples and controls were calculated by the cycle threshold method (ΔCt). The primer sequences used were as follows: β-actin, 5’- GAC GGC CAG GTC ATC ACT ATT G -3’ and 5’- AGG AAG GCT GGA AAA GAG CC -3’; Bhlhe41, 5’- TCG AAA CGG ACA GCC ATT GA -3’ and 5’- GAG CGC TCC CCA TTC TGT AA -3’; Bmi1, 5’- TGC AGA TGA GGA AA GAG GA -3’ and 5’- TCA TTC ACC TCT TCC TTA GGC -3’.

### Statistics

Data are presented as mean ± SEM. Statistical comparisons were performed using unpaired Student’s t-tests with GraphPad Prism 8 software (GraphPad Software Inc.). Correlation analysis was conducted using the Spearman rank correlation test. A two-tailed *P*-value of < 0.05 was considered statistically significant.

## Results

### B cell-specific deletion of *Bach2* reduces the number of B-1 cells in peripheral niches

**Figure 1:**
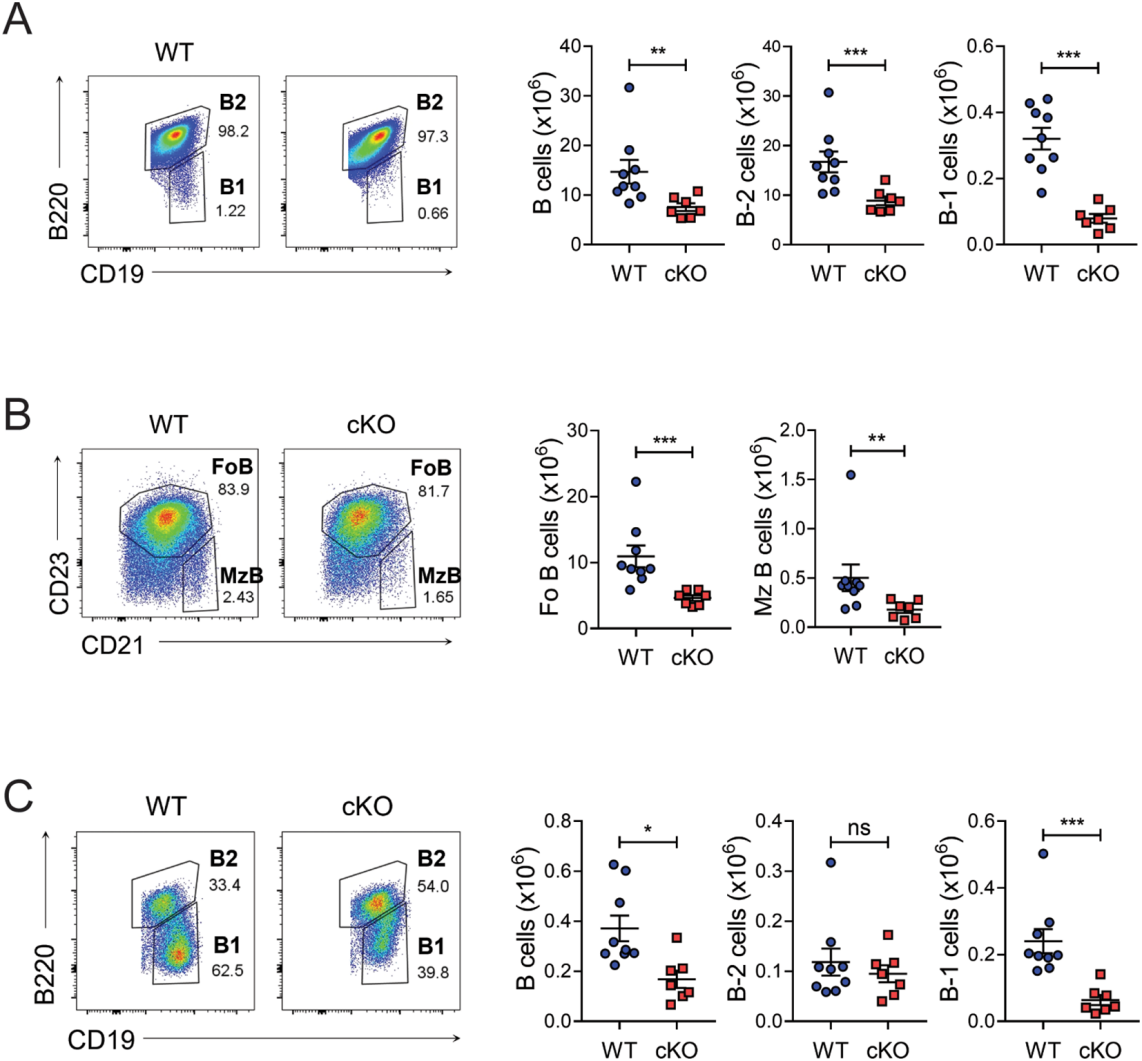
Reduced B-1 cell pools in B cell-specific Bach2-deleted mice. Spleen cells **(A, B)** and peritoneal lavage cells **(C)** from 6-week-old WT (n=9) and cKO (n=7) mice were analyzed by FACS. Representative FACS profiles gated on live CD19^+^ lymphocytes are shown. The graphs present means ± SEMs, with symbols representing the values of individual mice. The data are pooled from 3 independent experiments. *p < 0.05, **p < 0.01, and ***p < 0.001. by two-tailed unpaired Student *t*-test. FoB, follicular B; MzB, marginal zone B; ns, not significant.

To see whether a cell-autonomous function of Bach2 is required for the integrity of B cell populations, we generated a cKO strain in which *Bach2* was deleted specifically in CD19^+^ B-lineage cells (*Cd19^Cre/+^Bach2^fl/fl^*). Bach2 cKO mice contained normal numbers of pro-B, pre-B, and immature B cells in their BM, and numbers of B cells were also normal at transitional stages 1 to 3 in the spleen (**Supplementary Figure S1**). However, Bach2 deficiency affected numbers of B cells at the mature stage in the spleen, as well as numbers of recirculating mature B cells in the BM (**Figure 1**, **Supplementary Figure S1A**). Among mature splenic B cells, numbers of B-1 cells (CD19^+^B220^-^) in the cKO mice fell to approximately one quarter of those in their WT littermates, while numbers of whole B-2 cells (CD19^+^B220^+^), such as follicular (CD23^hi^CD21^lo^) and marginal zone B (CD23^lo^CD21^hi^) cells, fell to approximately half of those in their WT counterparts. We also assessed the distribution of B cells in the peritoneal cavity, which harbors the main niche for B-1 cells, and found that numbers of total B cells decreased significantly, largely due to a fall in B-1 cells but not B-2 cells (**Figure 1C**). The ratio of B-1a to B-1b cells did not differ between WT and cKO B-1 cells from either spleen or peritoneal cavity (data not shown). These results indicated that the B-1 cell pool was more substantially impaired by Bach2 deficiency than the peripheral mature B-2 cell pool.

### B-1 cell-specific Ab responses are reduced in Bach2 cKO mice

**Figure 2:**
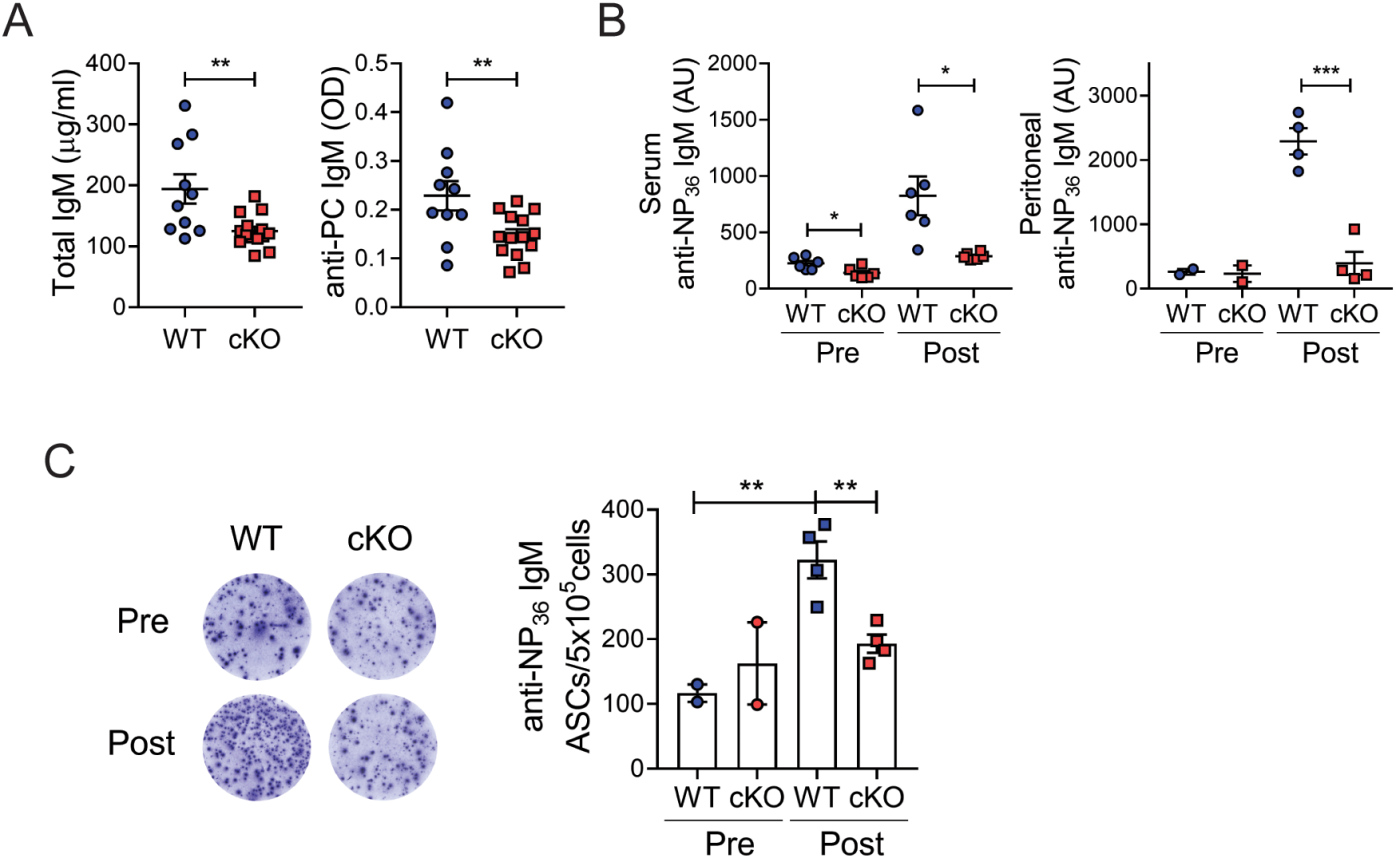
Reduced Ab responses of B-1 cells. **(A)** Sera from 6-week-old WT (n=10) and cKO (n=14) mice were assayed by ELISA to measure whole IgM and phosphorylcholine (PC)-specific IgM. **(B, C)** WT and cKO mice were immunized with NP-Ficoll and assayed *post mortem* on day 6 post-immunization. Titers of anti-NP_36_ IgM from sera and peritoneal lavage fluids were measured by ELISA **(B)**. Spleen cells were assayed by ELISPOT to determine numbers of NP_36_-specific IgM Ab-secreting cells (ASCs) per 5x10^5^ cells **(C)**. Representative wells are shown. The graphs present means ± SEMs values, with symbols representing the values of individual mice. The data are pooled from 2 independent experiments. *p < 0.05, **p < 0.01, and ***p < 0.001 by two-tailed unpaired Student *t*-test. AU, arbitrary unit; Pre, pre-immunization; post, post-immunization.

B-1 cells are the main source of natural IgM Abs, including autoantibodies against phosphorylcholine. Since numbers of B-1 cells were dramatically reduced in Bach2 cKO mice, we anticipated that natural Ab titers would also be lower. Indeed, **Figure 2A** shows that this expectation was borne out for the concentrations of whole and phosphorylcholine-specific IgM Abs.

B-1 cells also produce immune Abs upon challenge with T cell-independent (TI) antigens. We immunized Bach2 cKO and WT mice with NP-Ficoll, a type of TI antigen, and assessed the characteristics of NP-specific responses. After immunization, the titers of NP-specific IgM Abs in both the sera and peritoneal lavage fluids of WT mice were strongly elevated but this was not the case in cKO mice (**Figure 2B**). Since B-1 plasma cells migrate from their sites in local tissues to the spleen and BM, where they secrete IgM Abs (33), we compared numbers of ASCs in the spleen. Mirroring the ELISA result, anti-NP IgM ASCs were significantly less numerous in the spleens of cKO mice than of WT mice (**Figure 2C**). To determine which B cell subsets were responsible for this result, we fractionated follicular and marginal zone B cells, rather than B-1 cells due to their rarity, in both pre- and post-immunized mice. The marginal zone B cell fraction contained significantly fewer anti-NP IgM ASCs compared to the unfractionated cells, although it was slightly more abundant than the follicular B cell fraction (**Supplementary Figure S2**). Furthermore, the number of anti-NP IgM ASCs in the marginal zone B cells did not increase following immunization, suggesting that these ASCs are natural IgM-secreting cells, independent of NP-Ficoll immunization. Therefore, marginal zone B cells do not appear to be the primary producers of immune Abs following NP-Ficoll challenge in our system. Collectively, these results demonstrated that, consistent with the reduced numbers of B-1 cells, the Ab responses of B-1 cells are impaired in the Bach2 cKO mice.

### Bach2 deficiency reduces the size of fetal liver- and BM-derived B-1 cells populations

**Figure 3:**
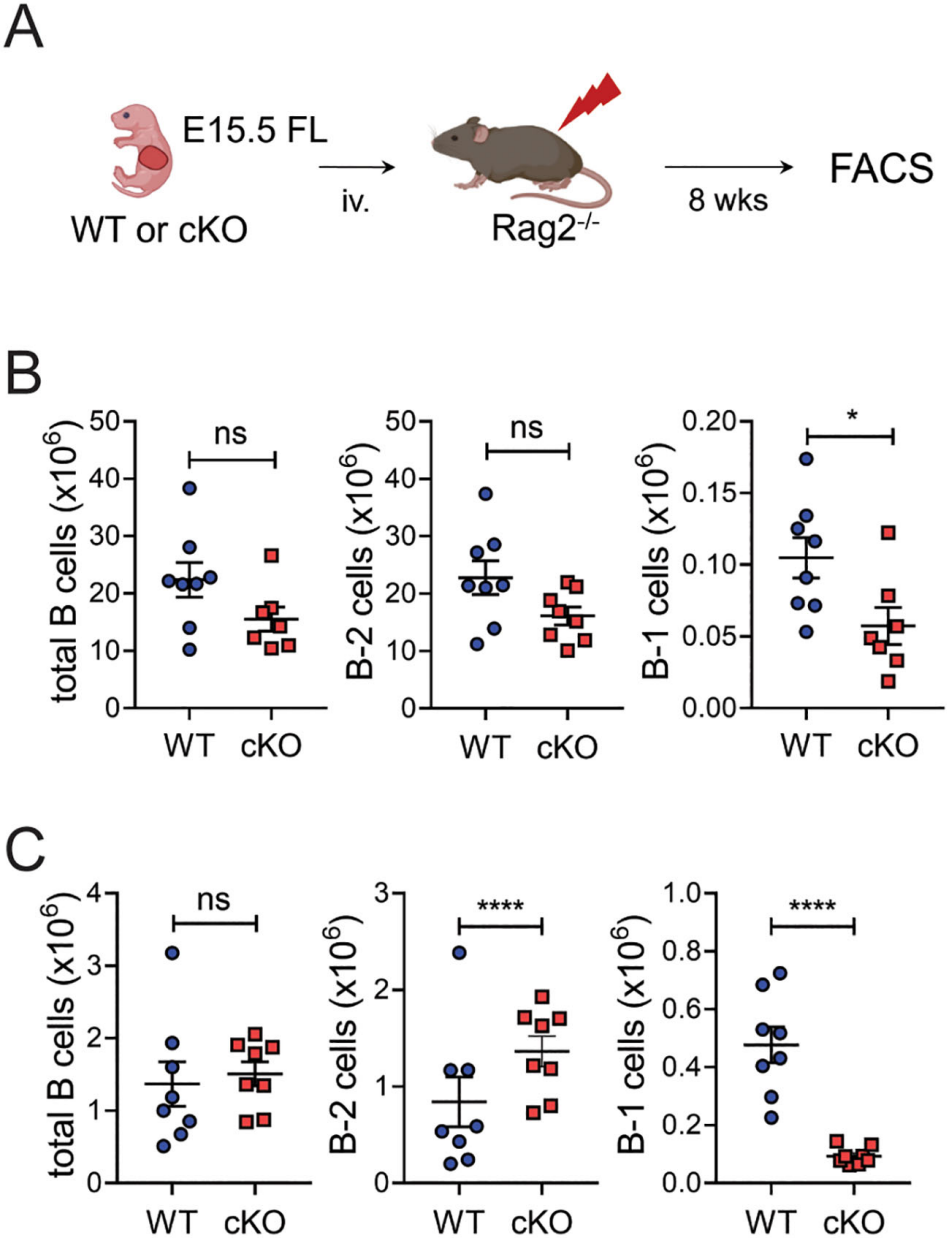
Bach2-deficient fetal liver cells have a specific defect in forming B-1 cell pools. Fetal liver cells from WT or cKO embryos on E15.5 were injected into *Rag2^-/-^* mice and assayed by FACS 8 weeks later (n=8/group). **(A)** Experimental scheme. **(B)** Numbers of total CD19^+^ B, B-2, and B-1 cells in the spleens of chimeric mice. **(C)** Numbers of total CD19^+^ B, B-2, and B-1 cells in the peritonea of chimeric mice. The graphs display means ± SEMs, with symbols representing the values of individual mice. The data are pooled from 3 independent experiments. *p < 0.05 and ****p < 0.0001 by two tailed unpaired Student *t*-test. ns, not significant.

Peripheral B-1 cells originate mainly from the fetal liver and are maintained thereafter by self-renewal (14, 15). The reduced B-1 cell pools in Bach2 cKO mice prompted us to ask whether Bach2 deficiency impairs the development of B-1 cells from the fetal liver. To this end, we generated chimeric mice by adoptively transferring fetal liver cells from cKO or WT mice into *Rag2^-/-^* mice and enumerated B cells at 8-weeks post-transfer (**Figure 3A**). The recipients of the cKO cells contained significantly fewer B-1 cells in both spleen and peritoneal cavity, whereas they contained similar or even higher numbers of B-2 cells (**Figures 3B, C**). This result demonstrated that Bach2 deficiency in fetal liver cells impairs their ability to form a normal B-1 cell pool in response to a normal evocative environment.

**Figure 4:**
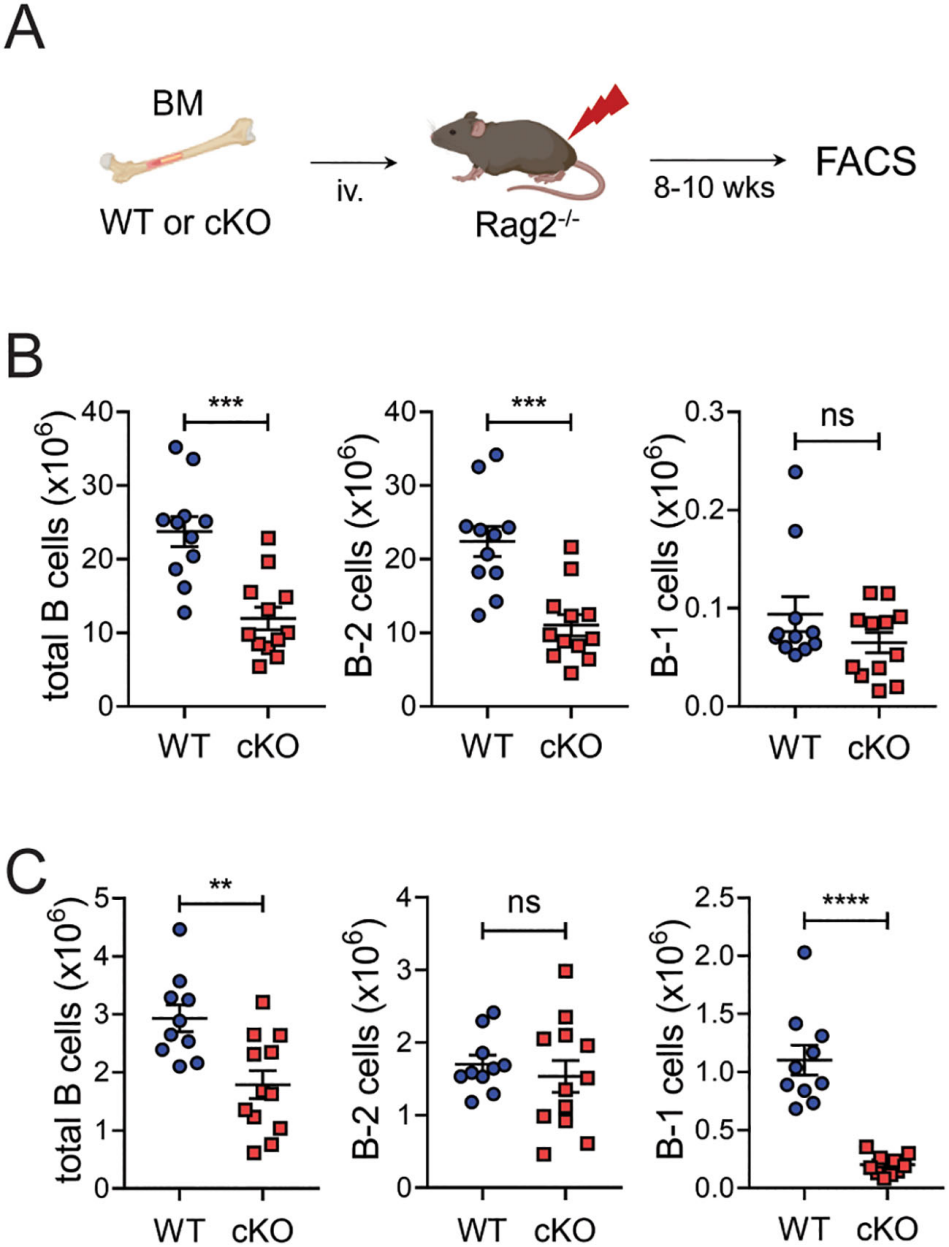
Impaired formation of peritoneal B-1 cell pools by Bach2-deficient BM cells. BM cells from WT or cKO mice were injected into *Rag2^-/-^* mice and assayed by FACS 8 weeks later (n = 12/group). **(A)** Experimental scheme. **(B)** Numbers of total CD19^+^ B, B-2, and B-1 cells in the spleens of chimeric mice. **(C)** Numbers of total CD19^+^ B, B-2, and B-1 cells in the peritonea of chimeric mice. The graphs present means ± SEMs, with symbols representing the values of individual mice. The data are pooled from 3 independent experiments. **p < 0.01, ***p < 0.001 and ****p < 0.0001 by two tailed unpaired Student *t*-test. ns, not significant.

A minor but substantial fraction of the B-1 cell population is derived from bone marrow progenitors (12, 13). To see whether Bach2 deficiency also affects the BM-derived B-1 cell pool, we generated another type of chimeric mouse by transferring BM cells instead of fetal liver cells into *Rag2^-/-^* mice (**Figure 4A**). This time the recipients of cKO cells contained dramatically reduced numbers of B-1 cells in their peritoneal cavities and slightly reduced numbers in their spleens (**Figures 4B, C**). However, in contrast to what happened with fetal liver cells, the cKO BM cells also gave rise to significantly fewer B-2 cells than WT BM cells in spleens but not peritonea.

These results taken together indicate that, regardless of their fetal liver or BM origin, the loss of Bach2 in fetal or hematopoietic cells impairs their ability to generate peritoneal B-1 cells. The loss of Bach2 during BM hematopoiesis seems to be more critical than at the fetal liver stage for establishing the B-2 cell pool.

### B-1 progenitor cells are not reduced in number in neonatal Bach2 cKO mice

**Figure 5:**
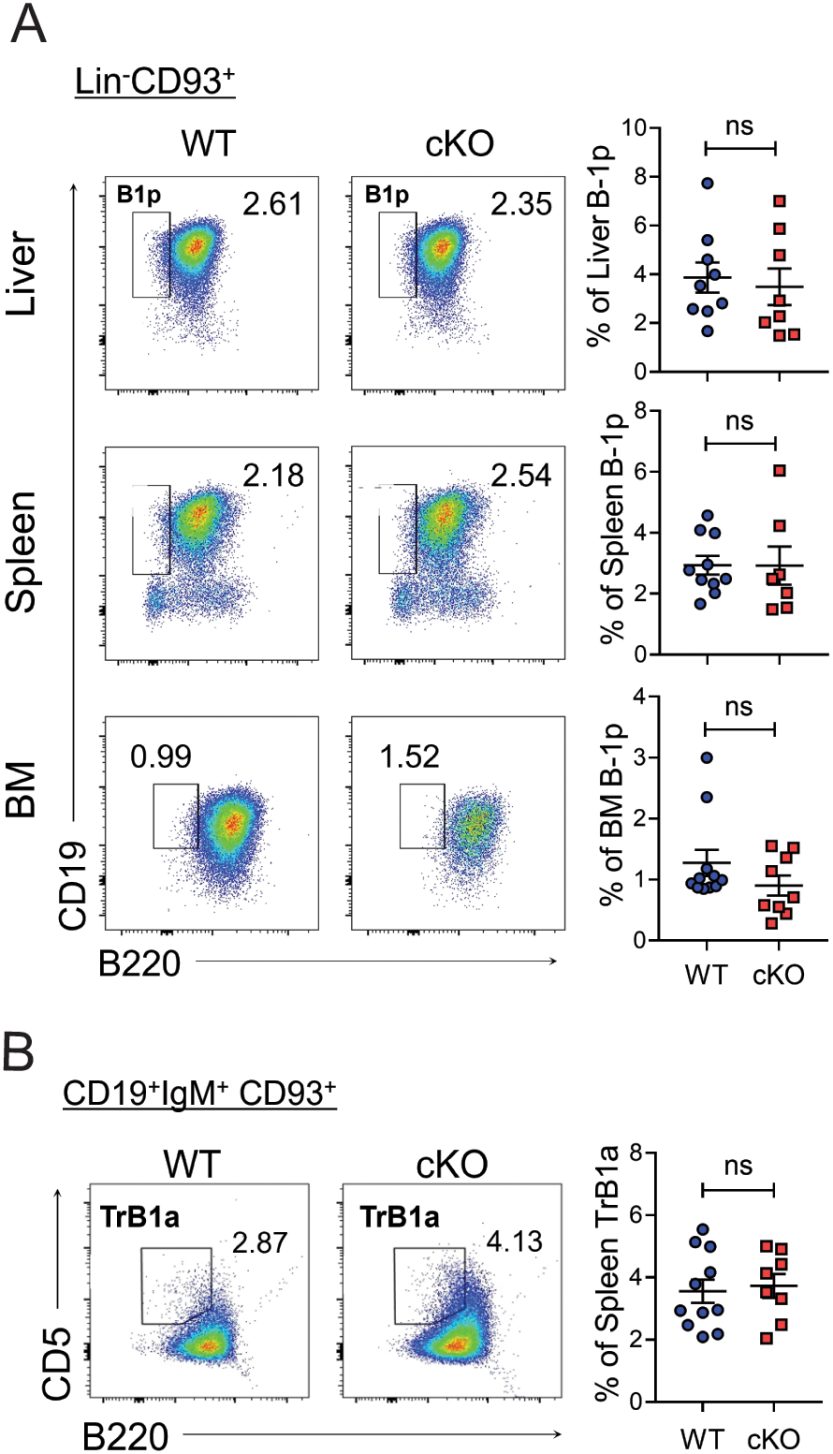
Normal distribution of Bach2-deficient B-1 progenitor cells. The liver, spleen, and BM were removed from WT and cKO mice on post-natal days 1 (liver and spleen) or 18 (BM) and assayed by FACS (n = 8-11/group). Representative FACS profiles with percentages of B-1 progenitor (B-1p) cells within Lin^-^CD93^+^ cells **(A)** and those of transitional (Tr) B-1a cells within splenic CD19^+^IgM^+^CD93^+^ cells **(B)** are shown. The data are pooled from 3 independent experiments. The graphs present mean ± SEM values. ns, not significant by Student t-test.

To determine whether Bach2 deficiency affects the early development of B-1 progenitors or their maturation, we assessed the proportion of B-1 progenitors— defined as Lin^-^CD93^+^B220^-^CD19^+^ cells (34, 35)— in neonatal mice. B-1 progenitor cells were detected in the liver and spleen on postnatal day 1 and in the BM on postnatal day 18. Importantly, the proportion of cells of this phenotype did not differ between WT and cKO mice in any of the three tissues (**Figure 5A**). A fraction of B cells with a CD19^+^IgM^+^CD93^+^B220^-^CD5^+^ phenotype has been identified as transitional B-1 progenitors in neonatal spleen (36). We found that the proportion of these progenitors again did not differ between WT and cKO mice at postnatal day 1 (**Figure 5B**).

These results demonstrate that Bach2 is dispensable for the early development of B-1-lineage cells up to the B-1 progenitor stage and therefore imply that post-progenitor maintenance of the B-1 cell pools requires a cell-autonomous function of Bach2.

### Bach2-deficient B-1 cells fail to be maintained by self-renewal in their peripheral niches

**Figure 6:**
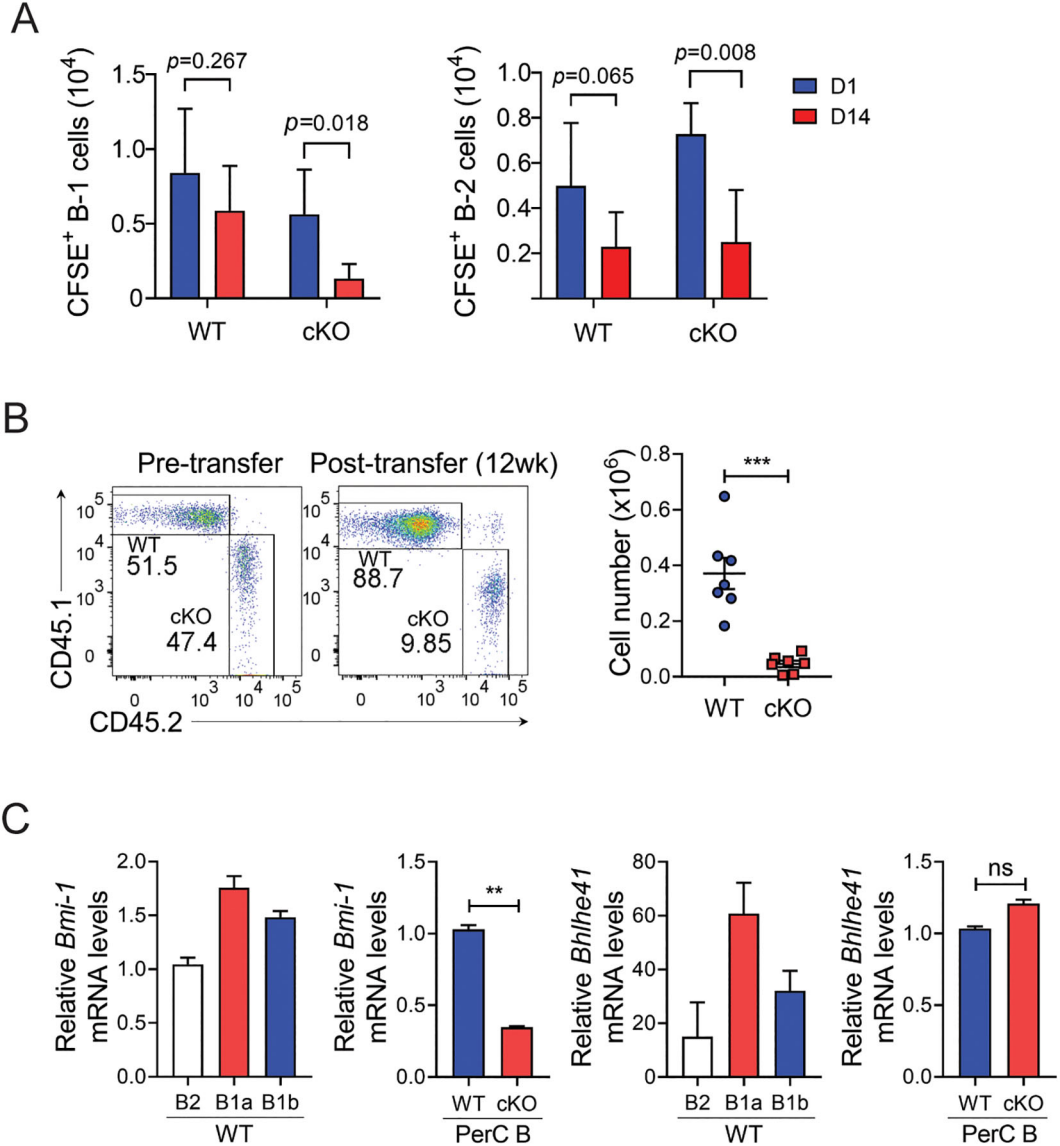
Impaired maintenance of mature Bach2-deficient B-1 cells. **(A)** Peritoneal CD19 B cells from WT or cKO mice were labeled with CFSE and injected intraperitoneal into WT mice. On day 1 and two weeks later, CFSE^+^ donor B cells were analyzed by FACS. The graphs display numbers of CFSE^+^ B-1 and B-2 cells in the peritoneal cavity as mean ± SEM values. **(B)** B-1a cells from CD45.1^+^ WT and CD45.2^+^ cKO mice were mixed in a 1:1 ratio and injected intraperitoneally into irradiated *Rag2^-/-^* mice. Twelve weeks after transfer, B-1a cells residing in the peritoneal cavity were analyzed by FACS. Representative FACS profiles gated on live cells are shown (n=7/group). Graphs display mean ± SEM numbers of CD45.1^+^ WT and CD45.2^+^ cKO B-1a cells 12 weeks after transfer. **(C)** CD19^+^ B cells were sorted from PECs of WT and Bach2 cKO mice by MACS, and WT B cells were further fractionated into B-2, B-1a, and B-1b cells by FACS. The cell fractions were assayed by quantitative RT-PCR. The relative mRNA expression of each gene was normalized with β-actin mRNA. The data are pooled from 2 **(A, B)** or 3 **(C)** independent experiments. **p < 0.01, and ***p < 0.001 by two tailed unpaired Student *t*-test. ns, not significant.

Unlike B-2 cells that are replenished continuously by BM hematopoiesis, B-1 cells maintain their levels by self-renewal in their peripheral niches. To determine whether Bach2 is required for this homeostatic mechanism, we adoptively transferred CFSE-labeled peritoneal B cells from cKO and WT mice into the peritonea of un-irradiated WT mice, and tracked CFSE^+^ donor cells on days 1 and 14 post-transfer. By day 14, the CFSE positivity among both WT and cKO B-1 donor cells had not been diluted (data not shown), indicating that the donor cells had not divide since their transfer. The total number of CFSE^+^ WT B-1 cells on day 14 was close to that on day 1, indicating that the WT B-1 cells had survived over that period (**Figure 6A**), whereas the total number of CFSE^+^ cKO B-1 cells had fallen sharply. Unlike WT B-1 cells, donor WT B-2 cells were not maintained in the recipient, as judged by an approximate halving of them by day 14, and there was a similar drop in the number of CFSE^+^ cKO B-2 cells. Thus, these results indicate that the absence of Bach2 results in a B-1 cell-specific failure of maintenance in their peripheral niche.

To evaluate the role of Bach2 in maintaining the mature B-1 cell population in more detail, we adoptively transferred a 1:1 mixture of CD45.1^+^ WT B-1a cells and CD45.2^+^ cKO B-1a cells into the peritonea of *Rag2^-/-^* mice. We found that 12 weeks after transfer the ratio of CD45.1^+^ to CD45.2^+^ B-1a cells was approximately 9:1, and the absolute number of CD45.1^+^ B-1a cells had increased approximately 3.5-fold, while CD45.2^+^ B-1a cells had approximately halved (**Figure 6B**). These results clearly demonstrated that Bach2-deficient B-1 cells were not maintained in the peritoneum whereas Bach2-replete B-1 cells effectively self-renewed after transplantation.

The transcription factors Bhlhe41 and Bmi1 have been identified as regulators of the development and/or self-renewal of B-1 cells (21, 22), and we confirmed that their expression in WT mice was higher in B-1a cells than in B-2 and B-1b cells. Importantly, the self-renewal factor Bmi1 was expressed at a lower level in cKO peritoneal B cells than in their WT counterparts (**Figure 6C**). In contrast, Bhlhe41 expression did not differ between WT and cKO peritoneal B cells. This result further supports our hypothesis that Bach2 deficiency affects the process of self-renewal of B-1 cells rather than their early development.

### The failure of self-renewal in Bach2-deficient B-1 cells is caused by a survival defect

**Figure 7:**
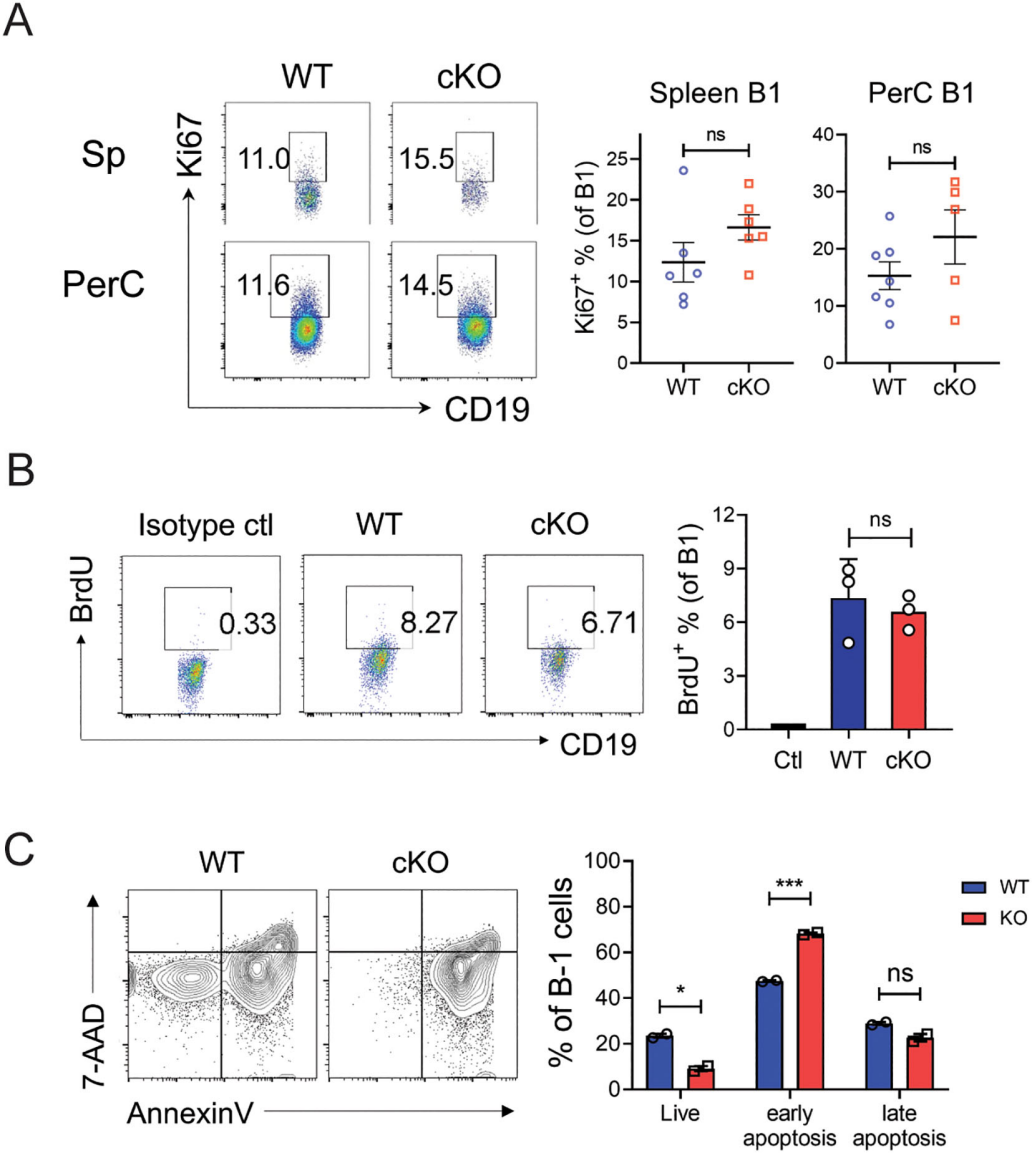
Impaired self-renewal of Bach2-deficient mature B-1 cells. **(A)** Spleen cells and peritoneal exudate cells from WT and cKO mice were stained with anti-Ki-67 antibody and assayed by FACS (n = 5-7 per group). Representative FACS profiles gated on CD19^+^B220^-^ and the percentages of Ki-67^+^ cells within B-1 cell fractions are shown. **(B)** WT and cKO mice were administered BrdU for 7 days and BrdU-incorporating cells were analyzed by FACS. Representative FACS profiles gated on CD19^+^B220^-^ and the percentages of BrdU^+^ cells within B-1 cell fractions are shown. **(C)** CD19^+^ B cells sorted from the peritoneal cavities of WT or Bach2 cKO mice were cultured without stimulators for 24 hours, stained with anti-CD19 and anti-B220 mAbs, Annexin V and 7-AAD and analyzed by FACS. Representative FACS profiles gated on CD19^+^B220^-^ B-1 cells are shown. The graph displays the percentages of Annexin V^-^ 7-AAD^-^ (live), annexin V^+^ 7-AAD^-^ (early apoptotic), and Annexin V^+^ 7-AAD^+^ (late apoptotic) cells among B-1 cells. The data are representative of 2 independent experiments. The bar graphs present mean ± SEM values. *p < 0.05 and ***p <0.001 by two-tailed unpaired Student *t*-test. ns, not significant.

The failure to maintain the B-1 cell pool might be due to a proliferation defect and/or a survival defect. To distinguish these possibilities, we assessed the proliferation rates of WT and cKO B-1 cells *ex vivo* without stimulation, to maximize fidelity to *in vivo* conditions. The proportions of Ki-67^+^ cells (indicating proliferative capacity) among the B-1 cells in spleen and peritoneum did not differ significantly between WT and cKO cells (**Figure 7A**). Similarly, BrdU incorporation assays showed that the proportions of dividing cells among cKO B-1 cells were comparable to those in WT B-1 cells (**Figure 7B**). These findings suggest that the reduced size of the Bach2-deficient B-1 cell pool is not due to a defect in proliferation.

Next, we examined whether Bach2-deficient B-1 cells were more susceptible to apoptotic death: we incubated peritoneal WT and cKO B-1 cells without any stimulation for 24 hours and analyzed them by FACS after staining with Annexin V and 7-AAD. The proportion of live (Annexin V^-^ 7-AAD^-^) cells was significantly lower in the cKO cells than the WT cells, and this difference was due to an elevated proportion of early apoptotic (Annexin V^+^ 7-AAD^-^) cells among the cKO cells (**Figure 7C**). These results indicate that Bach2-deficient B-1 cells are more susceptible to apoptotic death than WT B-1 cells.

## Discussion

We found in the present study that CD19^+^ cell-specific deletion of Bach2 reduced numbers of B-1 cells in peripheral niches, and reduced B-1 cell-specific Ab responses. Bach2-deficient fetal liver and BM cells failed to form normal-sized B-1 cell pools when adoptively transferred into *Rag2^-/-^* mice. Nevertheless, the number of B-1 progenitor cells in early neonatal cKO mice was not reduced, suggesting that Bach2 deficiency specifically affects post-progenitor development including self-renewal. Indeed, Bach2-deficient B-1 cells expressed reduced levels of the self-renewal factor Bmi1 and failed to self-renew in the peritonea of *Rag2^-/-^* hosts. Such a defect might be caused by increased cell death rather than proliferative disability. Thus, these results indicate that Bach2 is crucial for the maintenance of mature B-1 cell pools in their peripheral niches, but not for their development *per se* in the generative tissues.

Transcription factors known to participate in the development and maintenance programs of B-1 cells can be categorized into 2 types, according to their specificities. One includes the transcription factors whose activities are important for both B-1 and B-2 cells but have a more pronounced influence on B-1 cells, such as Ebf1, Oct2 and IκBNS (37–39). The other includes the transcription factors Arid3a, Bhlhe41 and Bmi1, whose functions are mostly restricted to B-1a cell development and/or maintenance (21, 22, 40). Bhlhe41 seems to execute broader roles than Bmi1, since it controls diverse features of B-1 cells, including their development, self-renewal and BCR repertoire, while Bmi1’s activity is restricted to regulation of self-renewal. In view of our finding that the absence of Bach2 perturbed both the B-1 and B-2 cell pools, Bach2 appears to be a factor of the first type. However, we also noted crosstalk between Bach2 and Bmi1, a factor of the second type. In this regard, Bach2 may employ diverse mechanisms; one common to both B-2 and B-1 cells and the other unique to B-1 cells. Of note, the result that Bach2 is associated with Bmi1 but not Bhlhe41 further supports the view that the role of Bach2 is in self-renewal rather than developmental processes.

How might Bach2 control the self-renewal of mature B-1 cells? One possibility might be related to the function of Bach2 in controlling regulators of cell cycle and apoptosis, as described in previous reports. In line with our finding, an anti-apoptotic function of Bach2 has been reported in non-lymphoid cells where Bach2 protected pancreatic β-cells from pro-inflammatory cytokine-induced apoptosis by repressing the pro-apoptotic factors JNK1 and BIM (41), and rescued skin fibroblasts from senescence-inducing signals by repressing the cell cycle inhibitor p21 (42). In contrast, in mature B-2 cells, Bach2 directly represses cyclin D3, which maintains the cells under cell cycle arrest at steady state (43). However, Bach2 promoted the survival and cell cycle progression of follicular B-2 cells in response to BCR crosslinking, in association with repression of genes that encode cyclin-dependent kinase inhibitors (e.g., p19 and p21) and induction of the anti-apoptotic factor Bcl-x_L_ (44). Therefore, Bach2’s action on cell cycle regulation seems to be context dependent. Moreover, B-1 cells may employ a mechanism distinct from that of B-2 cells, to execute cell cycle progression and survival, as evidenced by the fact that cyclin D2 is essential for the BCR-mediated proliferation of B-1 cells but not of B-2 cells (45).

The IL-5/IL-5R system is known to be indispensable for maintaining B-1 cell pools as it promotes their survival and homeostatic proliferation (46). This dependence appears to be unique to B-1 cells as IL-5Rα is constitutively expressed on all B-1 cells at steady state, but not on most B-2 cells. B-2 cells also only respond to IL-5 signals after priming with antigens or LPS, and as a result differentiate into IgA-secreting cells. We found that the levels of IL-5Rα did not differ between WT and cKO B-1 cells (not shown), suggesting the effect of Bach2 on B-1 cell self-renewal is irrelevant to IL-5 signaling.

In summary, we have shown here, to the best of our knowledge for the first time, that Bach2 plays a crucial role in maintaining B-1 cell pools by regulating self-renewal. This study thus provides new insight into how Bach2 controls the shape of innate-like humoral immunity.

## Data Availability

The original contributions presented in the study are included in the article/**Supplementary Material**. Further inquiries can be directed to the corresponding authors.
