# Supplementary figures and images for: The transcription repressor Bach2 is required for maintaining the B-1 cell population by regulating self-renewal
Source: Front Immunol. 2025 Mar 18;16:1553089. doi: 10.3389/fimmu.2025.1553089 (PMC11958198; doi:10.3389/fimmu.2025.1553089)

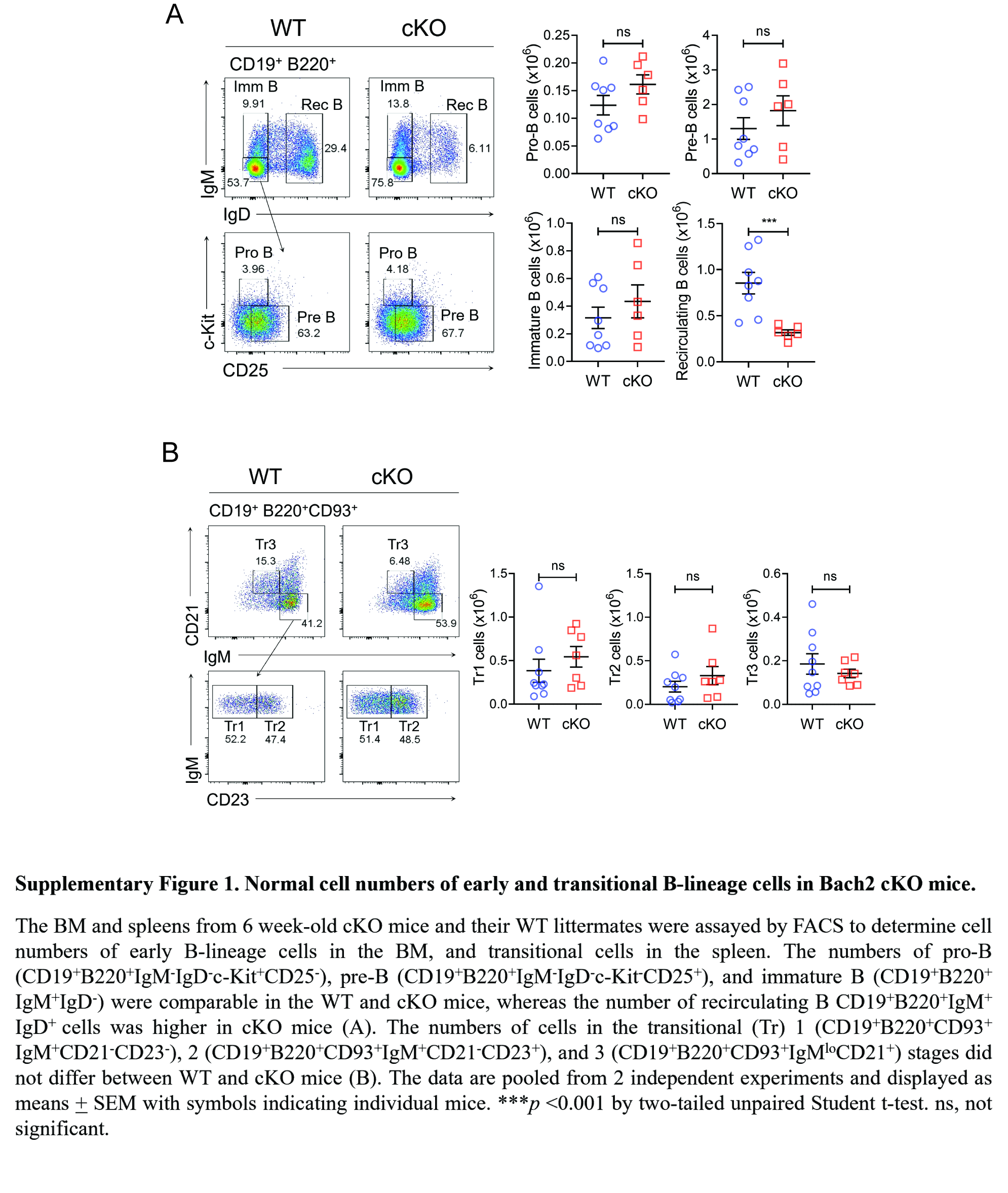

Supplement: Supplementary file 1 [file Image1.tif]

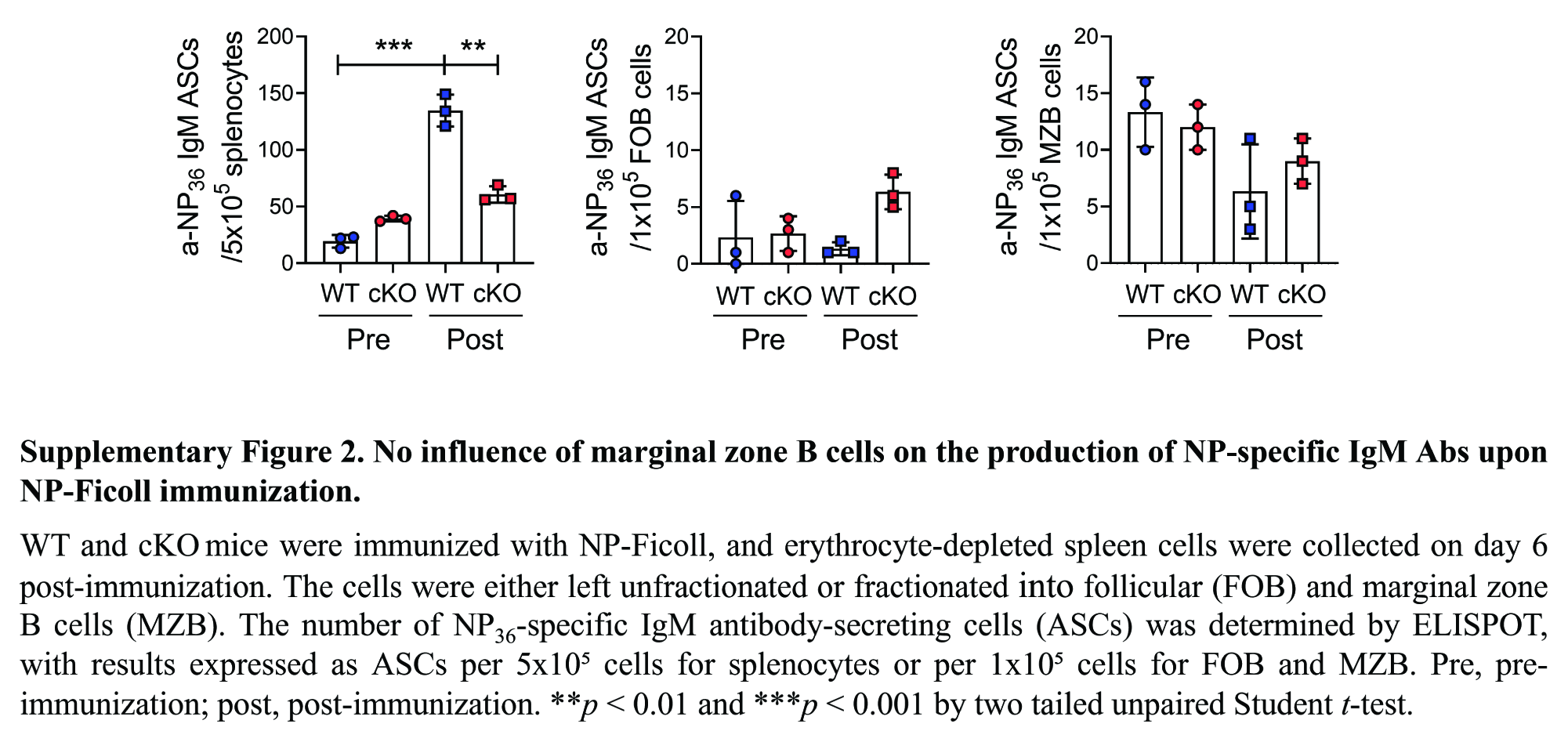

Supplement: Supplementary file 2 [file Image2.tif]
